# Seroprevalence of arenavirus and hantavirus in indigenous populations from the Caribbean, Colombia

**DOI:** 10.1590/0037-8682-0132-2019

**Published:** 2019-12-20

**Authors:** Amada Bolaños, Carolina Montoya-Ruiz, Juan Camilo Perez-Peréz, Juan David Rodas, Salim Mattar

**Affiliations:** 1 Universidad de Córdoba, Instituto de Investigaciones Biológicas del Trópico, Montería, Córdoba, Colombia.; 2 Universidad de Antioquia, Grupo Centauro, Medellín, Antioquia, Colombia.; 3 Universidad de los Andes, Laboratorio De Diagnóstico Molecular y Bioinformática, Bogotá D.C, Colombia.

**Keywords:** Health services, Indigenous population groups, Zoonoses, Rodent diseases, Arenaviruses, Hantaviruses

## Abstract

**INTRODUCTION:**

In Colombia, there is insufficient epidemiological surveillance of zoonotic hemorrhagic viruses.

**METHODS:**

We performed a sero-epidemiological study in indigenous populations of Wayuü, Kankuamos, and Tuchin communities using Maciel hantavirus and Junin arenavirus antigens for IgG detection by ELISA.

**RESULTS:**

IgG antibodies to hantavirus and arenavirus were found in 5/506 (1%) and 2/506 (0.4%) serum samples, respectively.

**CONCLUSIONS:**

Arenavirus and hantavirus circulate in indigenous populations from the Colombian Caribbean region, and the results indicate that the indigenous populations are exposed to these zoonotic agents, with unknown consequences on their health, despite low seroprevalence.

Epidemiological surveillance of hemorrhagic viruses in Colombia has focused primarily on Dengue, Zika, and Chikungunya, even though the geography and abundant biodiversity suggest that other zoonotic agents such as hantavirus and arenavirus could be important in terms of public health, especially in rural areas. Hantaviruses belong to the *Orthohantavirus* genus (*Hantaviridae* family) and are transmitted by shrews, moles, bats, and mice^1^. Hantavirsues transmitted by rodents are the most widely studied because they result in human disease. For example, hantavirus cardiopulmonary syndrome (HPS) is caused by viruses associated with *Cricetidae* rodents that circulate in the American continent^1^. Hantavirus circulation in Colombia was recently acknowledged through serological studies that detected hantavirus antibodies in humans and rodents from the Caribbean region^2^ ^-^ ^6^. Pathogenic arenaviruses in humans belong to the *Mammarenavirus* genus (*Arenaviridae* family), which is divided into two groups according to geographic distribution and antigenic characteristics: Lymphocytic choriomeningitis-Lassa and the Tacaribe complex. Some viruses from the Tacaribe complex cause severe hemorrhagic fevers in South America, such as Guanarito in Venezuela, Junín in Argentina, Machupo and Chapare in Bolivia, and Sabia in Brazil. All of these viruses are also hosted by rodents of the family *Cricetidae* ^7^. In Colombia, only Pichinde virus from the Tacaribe complex has been detected, but it has not been associated with any human disease^8^. 

The transmission of hantaviruses and arenaviruses through wild *Cricetidae* rodents suggests that people in rural areas, such as the indigenous inhabitants in the Caribbean area of Colombia, could be at risk of infection. In Colombia, it is well known that indigenous populations live in conditions of critical vulnerability and experience difficulties accessing health care services. The present study aimed to evaluate the seroprevalence of arenavirus and hantavirus in three indigenous communities located in the Colombia Caribbean region. 

We performed a cross-sectional study from August 2012 to May 2013 to find IgG antibodies against arenavirus and hantavirus in patients that attended a routine, voluntary examination at the health medical center. Populations came from three indigenous communities of Colombia; Wayuü in the department of Guajira, Kankuamos in the department of Cesar, and Tuchin in the department of Cordoba (Figure 1). The Wayuü community lives in the Guajira peninsula between the Colombian northern coast and the Venezuelan northwestern coast. At 180 m above sea level (masl), the region is characterized by a warm climate, with temperatures between 25 and 42ºC and 45% relative humidity. The Wayuü population has nomadic habits, and the inhabitants frequently move across the Colombian and Venezuelan borders. The indigenous community of Kankuamos is located in Cesar in the Colombian northeast. This area is located between 300 and 2,500 MASL, with an average temperature of 28ºC and a relative humidity between 60 and 75%. The Kankuamos population includes 12 communities and has an estimated population of 15,512 inhabitants. The majority of these people live in rural areas near Valledupar city, and their economic activities are farming and ranching on their own small properties. On the indigenous reservation of Tuchin, the population consists of mestizos and people who belong to various indigenous ethnic groups. Tuchin is located in the northeast of the Cordoba Department in the Colombian northwest, within an area of 128 km^2^ and an average temperature of 28ºC. The economic activities of these groups are agriculture and crafts (Figure 1). 

**FIGURE 1:**
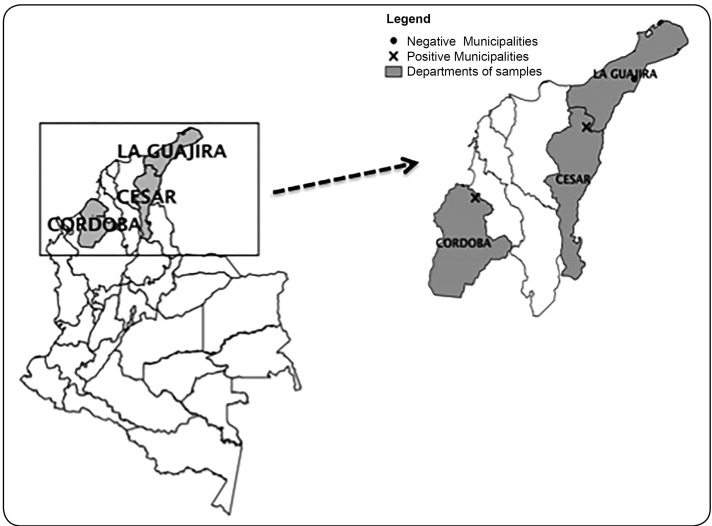
Geographical location of departments in which the sampled indigenous communities live.

The number of the samples from the Wayuü and Kankuamos communities was calculated according to the total number of indigenous people in each town and a probability of 0.5, with a confidence interval of 95% and a margin error of 0.8. Consequently, the number of the samples calculated for Wayuü was 171 and for Kankuamos was 167. Regarding the sample from the Tuchin town, the number of specimens was calculated according to the same parameters described above, except that the margin of error in this case was 0.07, resulting in 194 samples. However, we were able to obtain only 190 samples. 

Ethical guidelines were followed according to Resolution 008430 of October 4, 1993, of the Colombian Minister of Health and the Helsinki Declaration endorsed in 2004. Approval from the bio-ethical committee for this project was obtained through the Instituto de Investigaciones Biológicas del Trópico of the University of Córdoba, Monteria, which considered this as minimal risk research and approved the corresponding protocol and informed consent (number 006-2012).

Sampling was performed at the local health service for each community, where some of the people enrolled showed symptoms not necessarily related to hantavirus and arenavirus infection. All participants filled out an epidemiologic survey with personal data and data on ethnicity, household and geographic characteristics, occupation, the presence of rodents, and previous diseases compatible with hemorrhagic fevers. Serum samples were transported to the Universidad de Antioquia (Medellín, Colombia), where serological tests were performed. Detection of IgG antibodies to Maciel hantavirus (MCLV) and Junin arenavirus (JUNV) was carried out by ELISA using antigens donated by Instituto Nacional de Enfermedades Virales Humanas, Dr. Julio I. Maiztegui, INEVH (Pergamino, Argentina)^9^. Briefly, half of a 96-well plate was coated with Maciel or Junin antigens (infected-cells lysate) and the other half with a negative antigen (uninfected-cells lysate). Next, 100 µl of a 1:100 dilution of each serum sample was added to wells with positive and negative antigen, respectively. The antigen-antibody reaction was then detected using anti-human IgG plus alkaline phosphatase (KPL, Gaithersburg, United States) and TMB substrate (KPL, Gaithersburg, United States). Positive and negative controls were included in each plate, and the enzymatic reactivity was read at 450 nm. Samples with optical density (OD) higher than 0.2 at a dilution 1:400 were considered positive for both ELISA tests. The qualitative variables were expressed as absolute and relative frequency and the quantitative variables were expressed as mean with standard deviation. 

The Wayuü community was on average 31 years old and 80.2% female; 49% reported presence of rodents at home, 85% had a garbage collection service 2-3 times per week, and most individuals in the community were housewives (46%), students (20%), artisans (13%), or sellers (6%). The Kankuamos community was on average 33 years old and 59% female; 81% reported rodents at their home, 85% had garbage collection services every 3-4 days, and most individuals in the community were farmers (20%), housewives (22%), students (22%), or artisans (10%). Finally, the Tuchin population was an average 41 years old and 74% female. Only 30% confirmed seeing rodents at home, 62% had garbage collection services, and most individuals in the community were housewives (35%), artisans (25%), or students (16%). 

A total of 506 serum samples were evaluated, and 5/506 (1%) had IgG antibodies to MCLV and 2/506 (0.4%), to JUNV. Three of the MCLV positive samples were from the Kankuamos indigenous population, and two were from Tuchin (Table 1). The two JUNV-positive samples were from Kankuamos (Table 2). No positive samples were found in the Wayuü community.

**TABLE 1:** Results of individuals with IgG MCLV positivity on ELISA assay.

ID patient	ELISA Screening	Validation through titration (dilutions)		
	MCLV1/100	1/100	1/400	1/1600
Kankuamos 1	+	+	+	+
Kankuamos 2	+	+	+	-
Kankuamos 3	+	+	+	-
Tuchin 1	+	+	+	+
Tuchin 2	+	+	+	+

+: the titer of the serum was positive; -: the titer of the serum sample was negative. The cut off for the MCLV ELISA assay was OD 0.2 in a dilution of 1/400.

**TABLE 2:** Results of individuals with IgG JUNV positivity on ELISA assay.

ID patient	ELISA Screening	Validation through titration (dilutions)		
	JUNV 1/100	1/100	1/400	1/1600
Kankuamos 4	+	+	+	-
Kankuamos 5	+	+	+	-

+: the titer of the serum was positive; -: the titer of the serum sample was negative. The cut off for the JUNV ELISA assay is OD 0.2 in a dilution of 1/400.

The three Kankuamos males who had antibodies to MCLV confirmed seeing rodents in their home. Regarding their occupations, two were farmers and one was involved in social community activities. Garbage was not routinely collected in their neighborhoods. The construction materials used for all of their houses were wood and adobe, and the roof was made of palm leaves. It is remarkable that one IgG-positive patient had hypochondrium pain and that this patient showed the highest ELISA OD result (1/100 OD=1.8, 1/400 OD=0.97). Unfortunately, it was impossible to obtain a second serum sample from this individual to establish seroconversion and demonstrate a recent hantavirus infection. The other two IgG-positive indigenous males did not show any clinical symptoms compatible with HPS when interviewed at the health center. In the Tuchin group, a woman and a man had IgG antibodies to MCLV. The woman reported heart problems and muscular pain. Her house was built with cement, garbage was collected every three days, and she reported rodents in her home. Epidemiological data for the seropositive man were unavailable. Two Kankuamos women were IgG positive for JUNV, and both were involved in social community activities. They stated having seen rodents and other domestic animals in their houses, and the construction material of their houses was cement. 

This study evaluated exposure to roboviruses in three Colombian indigenous communities and found a 1% seroprevalence of Maciel virus and 0.4% seroprevalence of Junín. The hantavirus results we obtained are not very different from those of other studies performed in this country with a diverse population, especially those that used the same ELISA tests. A previous sero-survey in healthy, indigenous people from the de Emberá-Katío community located in the North of Antioquia reported a frequency of hantavirus and arenavirus infection of 1.5% and 3.1%, respectively^9^. Likewise, another study evaluated hantavirus and arenavirus antibodies in febrile patients from Urabá region (Antioquia) and reported a 0.5% seroprevalence of arenavirus infection and no hantavirus seropositive patients^10^. 

Although our study was not designed to recruit patients with specific symptoms for arenavirus hemorrhagic fever and HPS, it is remarkable that seropositive hantavirus infection in two people showed signs compatible with HPS. Nevertheless, we could not conclude that any of these symptoms correspond to a true hantavirus infection because we collected only one sample and were unable to perform differential diagnoses for other endemic pathogens that produce similar diseases. It is noteworthy that only one hantavirus infection case has been reported in Colombia, detected in the Cordoba department through IgM ELISA^5^.

Most health problems observed in the indigenous population may be due to economic and cultural factors; however, this population deserves more attention regarding the presentation of emerging infectious diseases because their nomadic lifestyle and frequent contact with wild animals could increase their exposure to zoonotic illnesses. Interestingly, our study suggests that one of the critical factors common to people exposed to rodent-borne diseases is garbage and waste material on the streets. Therefore, we believe that the very low seroprevalence could indicate either that robovirus infections truly present with a very low frequency or that some sub-clinical cases are overlooked. Both options are feasible for both viral families as infections ranging from those caused by non-pathogenic viruses to undifferentiated infections are detected, depending on the specific agent involved^1^ ^,^ ^7^.

Pichindé virus, hosted by *Oryzomys albigularis* from Pichindé Valley near Cali, Colombia, has been the only Tacaribe arenavirus species reported in Colombia^11^. This agent has been considered a non-pathogenic arenavirus, and its host distribution suggests that it is not in circulation in the area in which our study was performed^12^. However, according to the rodent distribution, it is possible that Guanarito virus, the etiological agent of Venezuelan hemorrhagic fever hosted by *Zygodontomys brevicauda,* is in circulation in this area^12^. Regarding hantavirus diversity, the only species reported in this region so far is Necoclí virus. However, its pathogenic potential is still unknown^13^.

In contrast, previous studies performed in different populations from surrounding areas and using different antigens found a higher seroprevalence. Mattar and Parra, for example, showed a hantavirus seroprevalence of 13.5% using Sin Nombre virus antigen in agricultural workers^2^. Guzman et al. found a seroprevalence of 3.5% and 7.3% for Maciel and Araquara viruses, respectively, in a similar population, but they used two different antigens^14^. We could speculate that a similar situation may have occurred with the low Junín arenavirus seroprevalence in this study because Junín is genetically far from Guaranito or Pichinde virus^11^. To test this hypothesis, we need to test other antigens such as Guanarito and Pirital viruses from Venezuela or Pichinde virus from Colombia.

In conclusion, our study shows arenavirus and hantavirus circulation in the indigenous population from the Colombian Caribbean region. The results are relevant and suggest to local and national health authorities that the government should invest more time and effort into studying emerging zoonosis, particularly in vulnerable and exposed groups with particular living conditions, such as those examined in this study.
